# Hypothyroidism in Thyroid Hemiagenesis: A Case Report

**DOI:** 10.7759/cureus.23087

**Published:** 2022-03-12

**Authors:** Rakesh Kumar Shah, Gagan Bohara, Fnu Juveria, Lubna Mirza

**Affiliations:** 1 Internal Medicine, Kathmandu Medical College and Teaching Hospital, Kathmandu, NPL; 2 Internal Medicine, Sahid Memorial Hospital, Kathmandu, NPL; 3 Internal Medicine, Deccan College of Medical Science, Hyderabad, IND; 4 Endocrinology, Norman Regional Hospital, Norman, USA

**Keywords:** anti-tpo antibody, autoimmunity, thyroid development, thyroid hemiagenesis, hypothyroidism

## Abstract

Thyroid hemiagenesis is one of the rare developmental abnormalities of the thyroid. It is more common in women and more commonly found on the left side, often associated with the absence of isthmus. In most instances, thyroid hemiagenesis is presented with hyperthyroidism. This case report aimed to present a 72-year-old female who presents with thyroid hemiagenesis and associated hypothyroidism. She was initially diagnosed with hypothyroidism about 25 years ago. There was no family history of thyroid disease, and she never underwent any neck, thyroid, or parathyroid surgeries. Her most recent laboratory investigations revealed thyroid-stimulating hormone level of 0.93 mIU/ml, free tetraiodothyronine of 0.93 mcg/dl, free triiodothyronine of 2.75 ng/dl, anti-thyroid peroxidase of 2.0 IU/ml, and thyroid-stimulating immunoglobulin of less than 1.0 IU/l. An ultrasound study of her neck revealed an absence of the left thyroid lobe and isthmus. The diagnosis of congenital thyroid hemiagenesis with hypothyroidism was made, and the current treatment with 75 mcg of levothyroxine was continued. Thyroid hemiagenesis is an incidental finding and may present later in life. Thyroid hemiagenesis is commonly associated with hyperthyroidism, but it can present with hypothyroidism. Patients with thyroid hemiagenesis may be at higher risk for developing hypothyroidism than their normal counterparts due to smaller thyroid hormone reserves.

## Introduction

Thyroid hemiagenesis (TH) is a rare disorder resulting from a failed embryological development of one thyroid lobe. Thyroid hemiagenesis develops more frequently on the left side for men in the ratio of 4:1 and women in the ratio of 3:1 [1]. Thyroid hemiagenesis is thought to be one of the rarest thyroid gland developmental defects, and the literature suggests that it affects 0.02% of the population [2]. The cause of thyroid hemiagenesis remains unknown, but there might be a genetic component to its etiology [3]. We present a case of congenital thyroid hemiagenesis and a review of relevant literature.

## Case presentation

A 72-year-old woman was referred to an endocrinology associate in Norman, Oklahoma, in August 2021 for hypothyroidism. She was diagnosed with hypothyroidism about 25 years ago and is taking 75 mcg of levothyroxine.

On presentation at the endocrine clinic, she complained of tinnitus, snoring, sleeping with multiple pillows, constipation, and joint stiffness. Her past medical history was non-contributory. She was pregnant only one time, and there was no history of miscarriage. Her past medical history includes carotid artery disease, diastolic dysfunction of the heart, hypertension, hyperlipidemia, gastroesophageal reflux disorder, and HER2 positive breast cancer on the right side. She had undergone right lumpectomy and sentinel node biopsy along with cholecystectomy, dilation and curettage, removal of basal cell carcinoma from the back, and a colonoscopy. There was no history of thyroid disorder in the family. She did not smoke but drank alcohol regularly. She took antacids, vitamin D3 supplements, anastrozole, aspirin, amlodipine, and atorvastatin. On presentation, her vitals were stable and physical examinations were unremarkable.

**Figure 1:**
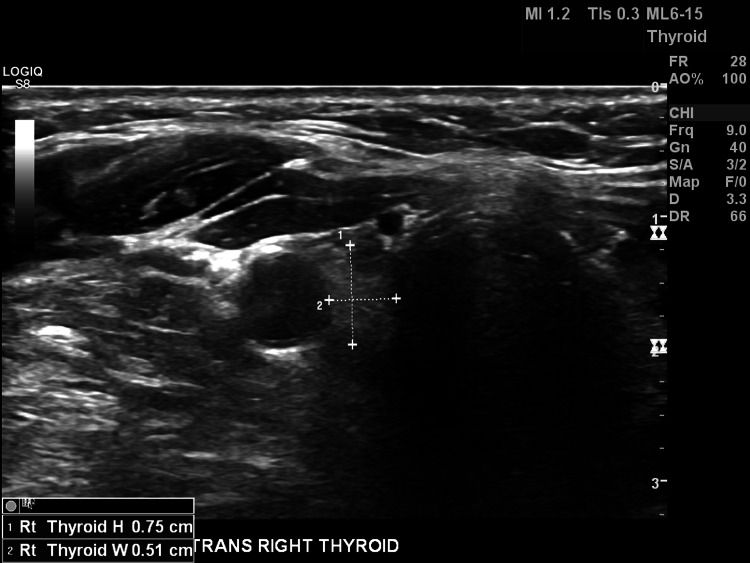
The ultrasound shows only the right lobe of the thyroid is present.

Recent laboratory investigations done in June 2021 are shown in Table 1. Free ultrasound of the thyroid showed a right lobe measurement of 0.75 cm x 1.13 cm x 0.51 cm. There was no evidence of compression, abnormal lymph nodes, or suspicious nodules. The left thyroid lobe and isthmus were not visible (Figure 1). She had not previously been told about this finding and had never undergone any thyroid surgery in the past. The diagnosis of congenital thyroid hemiagenesis and hypothyroidism without goiter was made. She was advised to continue 75 mcg of levothyroxine and follow-up with her primary care physician.

**Table 1:** Lab result of the patient (date of sample collection - June 2021).

S.N.	Test type	Result
1.	Tetraiodothyronine (T4)	0.93 mcg/dl
2.	Triiodothyronine (T3)	2.75 ng/dl
3.	Thyroid-stimulating hormone (TSH)	0.93 mIU/ml
4.	Thyroid-stimulating immunoglobin (TSI)	<1.0 IU/l
5.	Anti-thyroid peroxidase antibody (anti-TPO antibody)	2.0 IU/ml

## Discussion

Hemithyroid agenesis, a rare congenital thyroid defect, has been discovered to be more common in females by three folds [1]. Thyroid agenesis can be unilateral, complete, or isthmic [4]. In 80% of cases, the left lobe was missing, while in 20% of cases, the right lobe was missing with a left-to-right hemiagenesis ratio of 4:1. The isthmus may be lacking frequently [1]. The female patient, in this case, exhibited left-sided hemiagenesis as well.

In patients with thyroid hemiagenesis, the functioning lobe may experience pathological alterations comparable to those seen in a fully matured thyroid gland. There have been reports of hyperthyroidism, diffuse toxic goiter (Graves’ disease), toxic adenoma, toxic multinodular goiter, primary hypothyroidism, secondary hypothyroidism, chronic thyroiditis, colloid nodule, hyperparathyroidism, papillary carcinoma, and follicular carcinoma [1]. Compared to people with bilobate thyroid, patients with TH had a greater incidence of associated functional, morphological, and autoimmune thyroid problems [5].

Patients with TH, while usually clinically euthyroid, may present with significantly higher thyroid-stimulating hormone (TSH) and free triiodothyronine (FT3) [6]. It might suggest that the patient’s unilobed thyroid functional reserve has been compromised. There were no changes in free tetraiodothyronine (FT4) in some cases, while in some FT4 was also higher [5]. In our case, the patient's thyroid-stimulating immunoglobulins (TSI) are less than 1.0. She had no antibodies to thyroid peroxidase (TPO), and there was no compression, abnormal lymph nodes, or suspicious lumps seen on ultrasound. Thyroid pathology is more common in TH. Long-term TSH overstimulation is thought to be responsible for the high incidence of associated pathologies. TH diagnosis should be followed by careful surveillance and appropriate levothyroxine medication [5]. The patient's thyroid hormone levels fluctuated until she started levothyroxine 75 mcg. Now that her levels are normal, we'll need to continue with this dosage.

It's difficult to determine the true prevalence of thyroid hemiagenesis because the diagnosis is made in a population where other thyroid pathologies are being investigated [1]. TH is diagnosed using a combination of sonography and scinti scan, which is used to rule out the presence of functional thyroid tissue on the contralateral side of the lobe seen on ultrasonography and to see if there is any ectopically positioned accessory thyroid tissue [7]. In suspected cases like the unilateral absence of function in thyroid scintigraphy, an ultrasound examination can quickly confirm the diagnosis [8]. It was also used in our patients to diagnose thyroid hemiagenesis. The isthmus and left thyroid lobe were not visible. Our patient has never had surgery, therefore, TH would be congenital. Some patients with TH may benefit from levothyroxine treatment to lower the thyrotropin levels; however, this needs further research. Most of the patients with TH should be treated according to their associated pathology [9].

## Conclusions

Thyroid hemiagenesis can be an incidental finding and may be diagnosed only during late adulthood. It can be presented with both hyperthyroidism or hypothyroidism and can be associated with multinodular goiter, chronic thyroiditis, adenocarcinoma, and papillary thyroid carcinoma among other pathology. It is essential to educate patients about these conditions and provide appropriate follow-up if necessary.
